# Neurologic Alveolar Echinococcosis in Postpartum Zoo-Housed Gorilla, the Netherlands, 2024

**DOI:** 10.3201/eid3207.260136

**Published:** 2026-07

**Authors:** Laura A.N. Derks, Marieke Opsteegh, Denise Hoek-van Deursen, Jorrit J. Hofstra, Christine Kaandorp-Huber, Jooske IJzer, Erik A.W.S. Weerts, Volker H. Hackert, Anna R. Tellegen, Vanessa X.N. Visser, Joke W.B. van der Giessen

**Affiliations:** Centre for Infectious Disease Control, National Institute for Public Health and the Environment, Bilthoven, the Netherlands (L.A.N. Derks, M. Opsteegh, D. Hoek-van Deursen, J.J. Hofstra, J.W.B. van der Giessen); GaiaZOO, Kerkrade, the Netherlands (C. Kaandorp-Huber); Veterinary Pathology Diagnostic Centre, Utrecht University, Utrecht, the Netherlands (J. IJzer, E.A.W.S. Weerts); Public Health Service South Limburg, Heerlen, the Netherlands (V.H. Hackert); Faculty of Veterinary Medicine, Utrecht University, Utrecht (A.R. Tellegen); Dutch Food and Consumer Product Safety Authority, Utrecht (V.X.N. Visser)

**Keywords:** Echinococcosis, *Echinococcus multilocularis*, gorilla, parasites, *Gorilla gorilla*, primates, zoonoses, public health, pregnancy, the Netherlands

## Abstract

We report a case of postpartum alveolar echinococcosis in a zoo-housed gorilla in the Netherlands in 2024, with cerebral involvement causing neurologic symptoms. Infection was likely acquired via contaminated feed. This case highlights diagnostic challenges, public health risks, and the need for preventive feed hygiene and surveillance in endemic regions.

*Echinococcus multilocularis*, a zoonotic tapeworm with foxes as the main definitive host and rodents as intermediate hosts, was first detected in foxes in the Netherlands in 1996 (*1*) and is considered an emerging parasitic pathogen (*2*). Humans and other primates can be infected via contaminated food or fomites, risking potentially fatal alveolar echinococcosis (AE). We describe a case of neurologic AE in a postpartum, zoo-housed gorilla in the Netherlands, complicated by pregnancy and neonatal care.

In April 2024, a 25-year-old female western lowland gorilla (*Gorilla gorilla gorilla*), born in England and transferred to GaiaZOO (Kerkrade, the Netherlands) in 2013, gave birth to her second young. The gorilla had no prior health issues. After parturition, lethargy and intermittent anorexia developed, followed by intermittent neurologic symptoms in 1 arm (hemiplegia) and both legs (paraplegia).

Parasitologic and bacteriologic stool diagnostics yielded no results. Because the gorilla was caring for a newborn, we initially withheld anesthesia-requiring diagnostics and initiated empirical treatment for various differential diagnoses, including *Balamuthia mandrillaris* infection. When symptoms progressed, we performed abdominal ultrasonography and blood sampling under sedation, revealing abscess-like liver lesions. Fine-needle aspiration biopsy yielded bacteriologically and mycologically negative purulent material, negative for *B. mandrillaris* by PCR (Erasmus Medical Center, Rotterdam, the Netherlands). After blood tests and cytology yielded no diagnosis, we submitted serum for *E. multilocularis* serology (Laboklin, Bad Kissingen, Germany).

While awaiting results, we noted stiffness and worsening of hemiparesis in the gorilla. We postponed euthanasia for the newborn to learn to drink from a bottle and bond with another female gorilla. By August 2024, the infected gorilla was unable to walk, prompting euthanasia. Simultaneously, serologic results revealed *E. multilocularis* infection. Imaging and necropsy showed lesions in the brain (Figure 1, panel A; Figure 2, panel A), lungs (Figure 1, panel B), and liver (Figure 1, panel C; Figure 2, panel B). Molecular (12S and COX1) and serologic tests confirmed *E. multilocularis* infection, acquired after 2016 (Appendix), leading to a final diagnosis of disseminated alveolar echinococcosis with liver, lung, and cerebral lesions.

**Figure 1:**
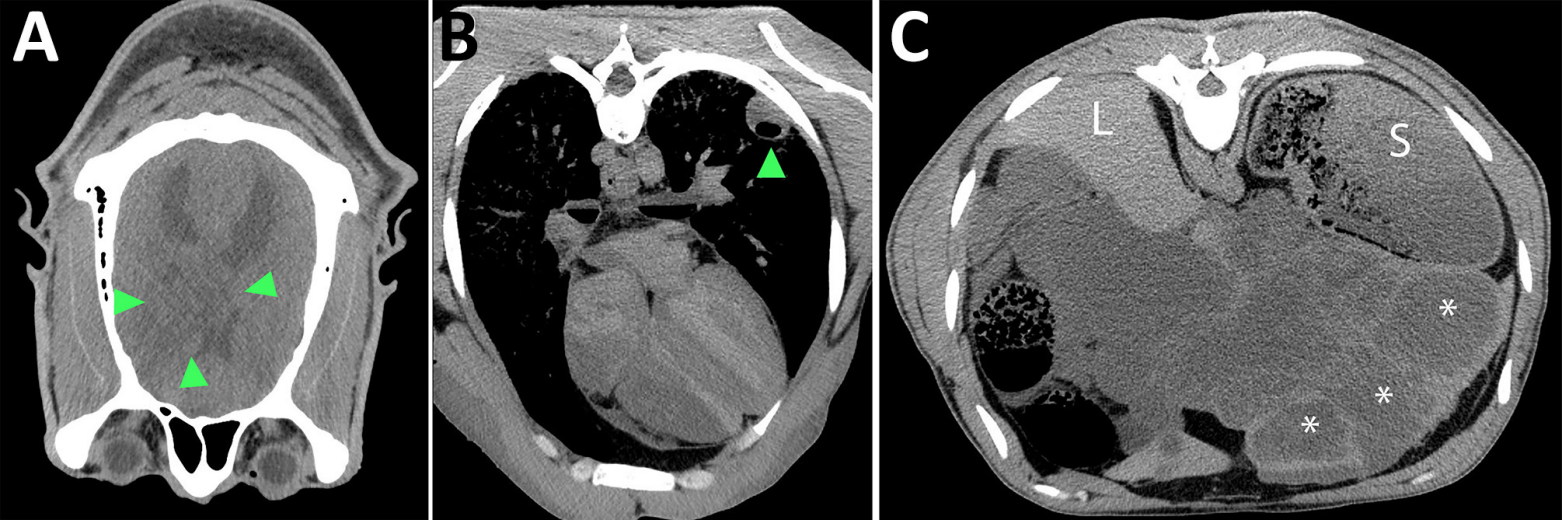
Postmortem computed tomography images in soft tissue window from a case of neurologic alveolar echinococcosis in postpartum zoo-housed gorilla, the Netherlands, 2024. A) Transverse view of the brain (anterior is bottom, right is left of image). Green arrows show space-occupying lesion within right hemisphere of cerebrum. B) Transverse view of the lung shows fluid- and gas-filled lesion within the left lung lobe (arrow). C) Transverse view of the liver shows multiple thick-walled, fluid-filled lesions (asterisks). L indicates normal liver tissue; S indicates stomach.

**Figure 2:**
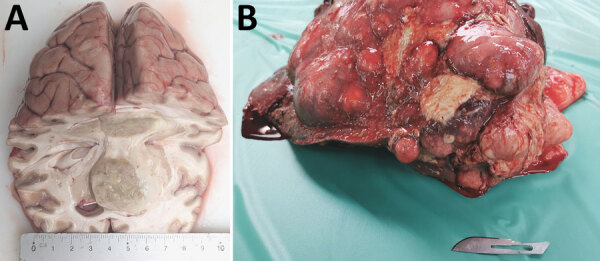
Necropsy brain and liver tissue from a case of neurologic alveolar echinococcosis in postpartum zoo-housed gorilla, the Netherlands, 2024. A) Cut surface of the cerebrum. Right hemisphere contains a 3.5-cm diameter, space-occupying tissue mass with ill-defined borders and secondary dislocation of preexisting structures. B) Surface of the liver. The parenchyma is largely replaced by multiple variably sized confluent nodules.

Prior reports have described AE in gorillas (*3*,*4*), noting clinical similarities of progressive apathy, anorexia, and fibrous abdominal adhesions (*3*–*5*). In the case we describe, disease progressed in 4 months from onset to marked deterioration, a more rapid course than the 2-year progression described in cases from Switzerland and Germany, both of which lacked neurologic involvement. A case involving neurologic symptoms was reported in a gorilla in Japan that died after 9 months (*4*). In gorillas, cerebral lesions seem to accelerate disease progression and could be considered a marker of terminal AE, similar to humans (*6*).

Reports of AE in humans have noted an association between brain metastasis, occurring in 1%–3% of cases (*6*), and immune suppression, which, depending on the host’s cellular immunity and cytokine profiles, increases the host’s susceptibility to infection and parasitic growth rate. Researchers reporting an AE case in a woman residing in a highly endemic region of China hypothesized pregnancy as a predisposing factor for rapid disease progression in humans, including brain metastasis (*7*). Another report noted rapid disease progression during pregnancy in a woman with cystic echinococcosis (*8*).

Born in England, a country free of *E. multilocularis* parasites, the gorilla we describe was housed in the Netherlands from 2013 and was still serologically negative in 2016, implying local infection. Foxes entering the enclosure seemed unlikely because of physical barriers; therefore, foodborne infection seemed plausible. The gorilla’s diet included locally grown fresh produce, leaves, and branches. A prior study investigating fruit from this endemic region found some to contain *E. multilocularis* DNA (*9*). In addition, research conducted in a zoo in Switzerland revealed fresh produce from the primate diet to be contaminated with fox-specific cestodes (*10*), suggesting contact between fox feces and primate feed.

Strategies to minimize infection risks associated with AE include feed hygiene measures, such as thermo-treatment of branches, hard vegetables, and fruits, and purchasing leafy and soft vegetables from nonendemic areas (*10*). Feed should be stored indoors, with minimal contact with the ground. Foxes should be kept out where possible, and fox feces on zoo grounds should be removed, particularly because feces from infected foxes also pose a zoonotic risk to visitors and employees. To monitor foxes roaming the GaiaZOO, zoo staff now regularly collect droppings found on zoo grounds, which are then sent out and tested for *E. multilocularis* parasites.

The rapid deterioration due to cerebral involvement in the gorilla we describe illustrates the aggressive course AE can take when the brain is affected and demonstrates the importance of including AE in the differential diagnosis of neurologic disease, especially in immunocompromised or pregnant individuals in endemic areas. Foodborne transmission from locally grown products seemed the most probable infection source, which implies a risk for humans consuming fresh produce from endemic areas. Education and preventive measures could minimize infection risks for zoo animals and other consumers. Our case highlights the clinical and ethical complexities of managing AE in zoologic settings and reinforces the need for surveillance and prevention strategies at the human–animal–environment interface.

Appendix Additional information for neurologic alveolar echinococcosis in postpartum zoo-housed gorilla, the Netherlands, 2024
