# Supplementary material for: Neurologic Alveolar Echinococcosis in Postpartum Zoo-Housed Gorilla, the Netherlands, 2024
Source: Emerg Infect Dis. 2026 Jul;32(7):1211–4. doi: 10.3201/eid3207.260136 (PMC13322421; doi:10.3201/eid3207.260136)
Supplement: Appendix — Additional information for neurologic alveolar echinococcosis in postpartum zoo-housed gorilla, the Netherlands, 2024 [file 26-0136-Techapp-s1.pdf]

Article DOI: http://doi.org/10.3201/eid3207.260136

EID cannot ensure accessibility for supplementary materials supplied by authors. Readers who have difficulty accessing supplementary content should contact the authors for assistance.

Neurologic Alveolar Echinococcosis in Postpartum Zoo-Housed Gorilla, the Netherlands, 2024

Appendix

Detailed overview of post-mortem diagnostics and results

Necropsy

Macroscopic changes at necropsy included a tissue mass in the brain and multiple confluent pulmonic and hepatic abscesses. Histological examination revealed encapsulated necropurulent inflammation containing parasitic remnants (Appendix Figure 1).

DNA extraction of liver, brain and lung tissue

Frozen liver, lung and brain samples were submitted to the National Reference Laboratory for Parasites at RIVM for molecular diagnostics. After 1 week of storage in −80°C, DNA was extracted using the DNEasy Blood and Tissue Kit (Qiagen, Hilden, Germany) according to the protocol for tissue samples, with the following adaptations: after overnight lysis, the samples were centrifuged in a microcentrifuge for 1 minute at maximum speed. Only the supernatant was used for further extraction. Additionally, DNA was eluted in a total of 100 µL elution buffer by repeating elution with 50 µL two times. A positive control (*E. multilocularis*-positive fox feces with a Cq of 28) and negative control (PCR-grade H_2O) were included in the DNA extraction.

qPCR

To detect and quantify *E. multilocularis* DNA in the test samples, an *E. multilocularis*-specific qPCR was performed, targeting a small fragment of the 12S gene. The forward and reverse primer and probe used for this qPCR were previously described by Knapp et al. (*1*). Similarly to what was previously described by Opsteegh et al. in 2010 (*2*), an internal

competitive amplification control was developed and used to identify PCR inhibition. PCR-grade H_2O was used as negative control and DNA extracted from an *E. multilocularis* worm used as positive control. Each sample was performed in a final volume of 20 µL, containing 10 µL Maxima Probe qPCR Master Mix (2X) (Thermo Fisher Scientific, Waltham, MA, USA), 1.9 µL of each primer (20 µM), 0.1 µL of both hydrolysis probes (20 µM) with compatible fluorochromes FAM and Cy5, and 5 µL of extracted DNA. All samples were tested in triplicate and in different dilutions (undiluted, 10-fold and 100-fold). The qPCR was run on a LightCycler 480 Real-Time PCR System (Roche Diagnostics, Rotkreuz, Switzerland). The qPCR program used was previously described by Knapp et al. (2014) (*3*). qPCR results were analyzed using Roche Diagnostics LightCycler 480 Software version 1.5.1.62 and expressed as quantitative cycle (Cq) numbers (Appendix Table).

COX1 PCR and sequencing

To obtain DNA for sequencing, a PCR targeting the mitochondrial cytochrome c oxidase subunit I (COX1) was performed (*4*). Each assay was performed in a final volume of 55 µL, containing 5 µL DNA as well as 24 µL H2O, 0.5 µL of both primers (0,5 µM), and 25 µL HotStarTaq DNA Polymerase (Qiagen, Hilden, Germany). The PCR was run on a PTC Tempo 96 Thermal Cycler (Bio-Rad, Hercules, CA, USA). After an initial step of 15 minutes at 95°C, 35 cycles of 1 minute at 95°C followed by 1 minute at 45°C and 1:15 minutes at 72°C were repeated, with a final step of 10 minutes at 72°C. Positivity of the samples was checked using a FlashGel System (Lonza Bioscience, Basel, Switzerland). Cleanup of the PCR product was done using ExoSAP-IT PCR Product Cleanup Reagent (Applied Biosystems by Thermo Fisher Scientific), where 5 µL of PCR product was added to 2 µL ExoSAP-IT and incubated at 37°C for 15 minutes to degrade remaining primers and nucleotides. Next, the mix was incubated at 80°C for 15 minutes to inactivate the ExoSAP-IT reagent. For both the forward and reverse primer, a final volume of 20 µL, containing 5 µL of DNA, 5 µL of the primer (5 µM) and 10 µL H2O, was sent to BaseClear (Leiden, the Netherlands) for species determination by Sanger sequencing. The assembled sequence, with primer regions removed, was submitted to GenBank under accession number PZ227139 and analyzed using Chromas combined with NCBI BLAST and against our own reference sequences in BioNumerics version 8.1.1. Phylogenetic analysis showed high genetic similarity to other *E. multilocularis* isolates from Europe, including one from a red fox from the same region as the zoo where the gorilla was housed (Appendix Figure 2).

Serology

For detection of IgG antibodies against *E. multilocularis* in serum, *Echinococcus multilocularis* (Em2-Em18) IgG ELISA (Bordier Affinity Products, Crissier, Switzerland) with human conjugate was used according to protocol. To confirm serologic results, EUROLINE-WB Anti-*Echinococcus* (IgG) (Euroimmun, Lübeck, Germany) western blot was used according to protocol. Both ELISA and western blot confirmed seropositivity, which, combined with a negative 2016 serum, indicated infection was acquired after 2016. Though these serologic methods are intended for use on human samples, serum of the gorilla with AE gave a clear positive result. To investigate further exposure to *E. multilocularis*, other available gorilla sera were tested and found negative. However, these samples did not include all gorillas housed at the zoo, as blood sampling requires general anesthesia. Serum from remaining gorillas will be collected opportunistically when anesthesia is needed for future diagnostics or interventions.

References

1. Knapp J, Umhang G, Poulle M-L, Millon L. Development of a real-time PCR for a sensitive one-step coprodiagnosis allowing both the identification of carnivore feces and the detection of *Toxocara* spp. and *Echinococcus multilocularis.* Appl Environ Microbiol. 2016;82:2950–8. https://doi.org/10.1128/AEM.03467-15

2. Opsteegh M, Langelaar M, Sprong H, den Hartog L, De Craeye S, Bokken G, et al. Direct detection and genotyping of *Toxoplasma gondii* in meat samples using magnetic capture and PCR. Int J Food Microbiol. 2010;139:193–201. https://doi.org/10.1016/j.ijfoodmicro.2010.02.027

3. Knapp J, Millon L, Mouzon L, Umhang G, Raoul F, Ali ZS, et al. Real time PCR to detect the environmental faecal contamination by *Echinococcus multilocularis* from red fox stools. Vet Parasitol. 2014;201:40–7. https://doi.org/10.1016/j.vetpar.2013.12.023

4. Bowles J, Blair D, McManus DP. Genetic variants within the genus *Echinococcus* identified by mitochondrial DNA sequencing. Mol Biochem Parasitol. 1992;54:165–73. https://doi.org/10.1016/0166-6851(92)90109-W

Appendix Table. Results of *E. multilocularis*-specific qPCR of liver, lung, and brain samples. For the liver samples, a distinction is made between the wall and the contents of the abscess-like lesion. The average Cq and range of Cq values is displayed for each sample and dilution.

Tissue	Cq (undiluted)	Cq (10× diluted)*	Cq (100× diluted)*
Liver (lesion wall)	22.28 (21.27–23.30)	19.55 (19.54–19.55)	21.79 (21.78–21.79)
Liver (lesion content)	37.14 (34–28–40.00)	18.57 (18.56–18.57)	20.56 (20.53–20.59)
Lung	28.38 (28.35–28.40)	31.77 (31.61–31.97)	34.69 (34.21–35.22)
Brain	18.00 (17.93–18.09)	20.96 (20.90–21.01)	24.39 (24.33–24.49)

* DNA was diluted 10× and 100× in Milli-Q water to account for potential qPCR inhibition due to a too large quantity of DNA.

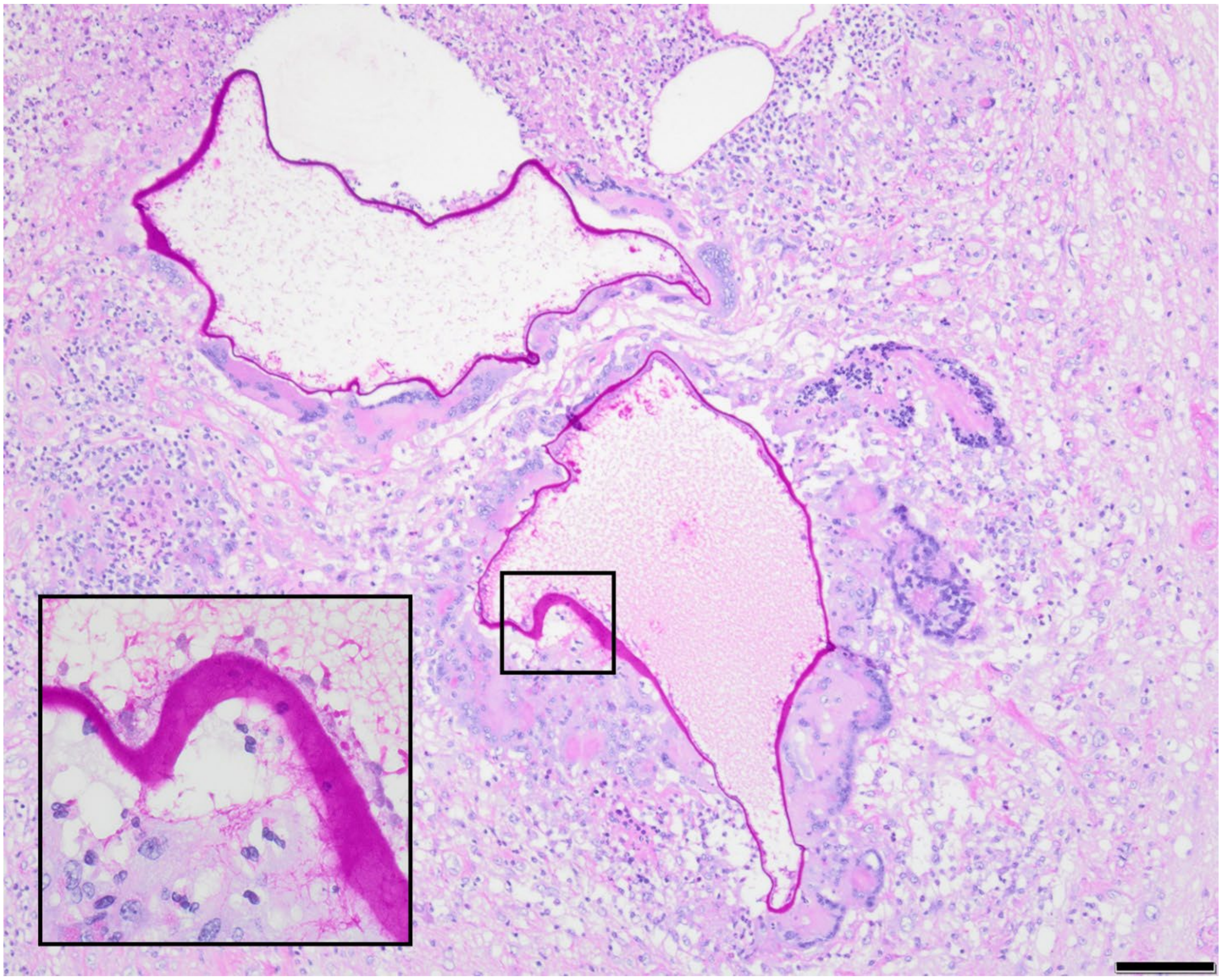

Appendix Figure 1. Cerebrum, microscopy. PAS staining, original magnification 100×, bar = 100 micron. Two collapsed parasitic remnants visible as slender laminated layers, with strong purple (positive) staining. Surrounding strong inflammatory reaction consists of large multinucleated giant cells, numerous smaller neutrophils located in lytic necrosis (malacia). Intact protoscolices were absent. **Inset**. Cerebrum, microscopy. PAS staining, original magnification 400×. Cyst wall is slender and does not show any obvious striation, consistent with alveolar echinococcosis.

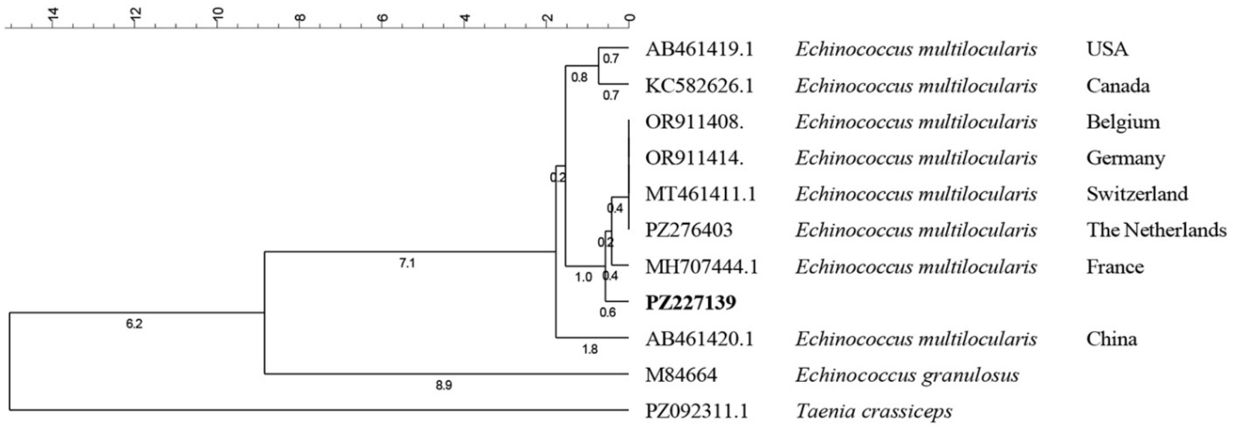

Appendix Figure 2. Phylogenetic tree of the *E. multilocularis* isolate from the gorilla (PZ227139, in bold) compared to reference COX1 sequences from Europe, North America, and Asia. *Echinococcus granulosus* and *Taenia crassiceps* were included as outgroups. The scale bar and branch labels represent the percentage of nucleotide divergence between the COX1 sequences. Reference sequences were downloaded from GenBank; accession numbers are provided. Alignment was performed in BioNumerics version 8.1.1.
